# No ground truth? No problem: Improving administrative data linking using active learning and a little bit of guile

**DOI:** 10.1371/journal.pone.0283811

**Published:** 2023-04-04

**Authors:** Sarah Tahamont, Zubin Jelveh, Melissa McNeill, Shi Yan, Aaron Chalfin, Benjamin Hansen

**Affiliations:** 1 Department of Criminology and Criminal Justice, University of Maryland, College Park, MD, United States of America; 2 College of Information Studies, University of Maryland, College Park, MD, United States of America; 3 Crime Lab New York, University of Chicago, New York, NY, United States of America; 4 School of Criminology and Criminal Justice, Arizona State University, Phoenix, AZ, United States of America; 5 Department of Criminology, University of Pennsylvania, Philadelphia, PA, United States of America; 6 National Bureau of Economic Research, Cambridge, MA, United States of America; 7 Department of Economics, University of Oregon, Eugene, OR, United States of America; Vellore Institute of Technology: VIT University, INDIA

## Abstract

While linking records across large administrative datasets [“big data”] has the potential to revolutionize empirical social science research, many administrative data files do not have common identifiers and are thus not designed to be linked to others. To address this problem, researchers have developed probabilistic record linkage algorithms which use statistical patterns in identifying characteristics to perform linking tasks. Naturally, the accuracy of a candidate linking algorithm can be substantially improved when an algorithm has access to “ground-truth” examples—matches which can be validated using institutional knowledge or auxiliary data. Unfortunately, the cost of obtaining these examples is typically high, often requiring a researcher to manually review pairs of records in order to make an informed judgement about whether they are a match. When a pool of ground-truth information is unavailable, researchers can use “active learning” algorithms for linking, which ask the user to provide ground-truth information for select candidate pairs. In this paper, we investigate the value of providing ground-truth examples via active learning for linking performance. We confirm popular intuition that data linking can be dramatically improved with the availability of ground truth examples. But critically, in many real-world applications, only a relatively small number of tactically-selected ground-truth examples are needed to obtain most of the achievable gains. With a modest investment in ground truth, researchers can approximate the performance of a supervised learning algorithm that has access to a large database of ground truth examples using a readily available off-the-shelf tool.

## Introduction

One of the more exciting recent developments in empirical social science research is the increasing availability of large administrative databases and the ability to link across them to generate new insights. Indeed linked datasets have allowed researchers working in descriptive, predictive, and causal modalities to generate systematic inferences about large numbers of individuals [1–9]. Unfortunately, most administrative datasets are not designed to be linked to others and thus have no common and reliable unique identifiers. Most often, researchers must attempt to link individuals using their demographic characteristics, such as name, date of birth, race, gender, and address. Typically, only a subset of this information is available and the data are subject to missingness, transcription errors and other systematic imperfections [10, 11]. Accordingly, the linking process is often difficult and fraught with error, particularly in domains like criminal justice and social service provision where the manner in which the data are collected makes them especially susceptible to recording errors [12–14]. Machine learning researchers have developed techniques such as self-supervised learning [15] and source free domain adaptation [16] to address the problem of data linking with little to no access to ground truth labels; however, the implementation of these techniques generally requires technical expertise that is uncommon among applied social science researchers who are, nonetheless, interested in linking data without common unique identifiers.

An old adage in data analysis and statistical modeling highlights the self-evident but foundational principle that empirical estimates from a model are only as good as the underlying data used to generate them. In the data linking context, as researchers make errors in linking between administrative datasets, the analytic samples used to generate estimates of interest will also contain errors. Unfortunately, the consequences of even modest linking errors can be severe. Random linking errors lead to attenuated estimates of a treatment effect of interest [11]. While attenuation bias may not lead to important qualitative distortions in reasonably large datasets, in the many applications in which statistical power is constrained by the cost or availability of data—including, notably, the vast majority of randomized field experiments—the consequence will be an increase in the probability of a false negative or a Type II error. Given the tendency for underpowered studies to end up in the “desk drawer,” Type II errors can have devastating consequences not only for individual research studies but also for entire literatures [17–19].

Recognizing the fundamental importance of data linking to applied research, statisticians and computer scientists have, for many years, worked to develop probabilistic record linkage algorithms which use statistical patterns in identifying characteristics to perform linking tasks [20–22]. This research has established that probabilistic linking algorithms, in which cases are considered to be a match when they exceed some threshold probability of being a true link, outperform the seemingly “conservative” approach of exact matching [11]. Consequently, a great deal of work aims to improve the performance of such algorithms. Indeed, in recent years, approaches to linking have become ever more sophisticated [15, 16].

While, in principle, many of these algorithms do not require prior information on which two records refer to the same person, *supervised learning* algorithms—the most powerful approach to data linking—require a pool of “ground-truth” data in order to train the algorithm [23]. With a large enough training sample, the algorithm can learn from patterns in the data and deduce which records are likely to match one another in unseen data. Unfortunately, ground truth data is often unavailable and the cost of obtaining ground truth examples can be high, often requiring a researcher to manually review pairs of records to make an informed judgement about match status. Several recently published application examples involving name matching algorithms demonstrate the benefits of data linkage using ground truth labels [21, 24, 25]. Nevertheless, the time-consuming nature of the task makes it prohibitively costly to generate a large volume of ground-truth examples to improve algorithmic performance, if none already exist. Although there are efforts to reduce the cost of ground-truth discovery by crowdsourcing [26], this approach is not applicable to all kinds of datasets and research contexts.

An alternative approach to data linking is to use an *active learning* algorithm. Like supervised learning, the goal is to generate ground truth labels upon which the linking algorithm can be trained. However, the approach is far more surgical. Whereas supervised learning requires that the research team have access to a relatively large ground truth database, active learning algorithms request labels from the researcher for high leverage cases and learn from a much smaller number of ground truth labels [27–29]. These approaches typically work by presenting the human labeler with examples to label, starting with the example whose label would yield the most information. The researcher can label as many or as few examples as they want, using either their own time constraints or the diminished utility of labeling an additional example as a stopping criteria. While this approach is thought to be an improvement over unsupervised linking methods that do not use ground truth at all, the consequences on linking quality have not been rigorously analyzed. In particular, we may be concerned that the number of ground-truth examples needed to maintain linking performance below a particular maximal error rate might grow as a function of the size of the data sets and the relative frequency of mismatches between the data sets involved. Simply put, the concern is that the potential benefits of an active learning approach are necessarily limited by the paucity of ground truth data that active learning algorithms can access.

In this paper, we investigate the value of ground-truth examples for linking performance. Using a popular open-source record linkage algorithm, *dedupe* [30], we explore how the payoff to investing in ground truth labels varies based on linking context, such as the difficulty of the linking task, the size of the training dataset, and the size of the datasets involved in the linking task itself. Specifically, we fix the share of true matches that do not match exactly on name and date of birth at 50 percent (which is likely at the “messier” end of data sets we have observed in the field) and vary the following parameters to explore the responsiveness of the linking error rate to the difficulty of the matching task: the size of data set that was used to train an algorithm and the size of the pool of potential records.

Our principle contributions are as follows:

1. We provide a roadmap for applied social science researchers—especially those who are unfamiliar with the technical data science literature on record linking—to perform data linking efficiently when ground truth information is unavailable.
2. We confirm the common sense finding that data linking can be dramatically improved with the availability ground truth examples. But critically, we note that in many applications, only a relatively small number of tactically-selected ground-truth examples are sufficient to obtain most of the achievable gains.
3. We show, that in the context of a common linking task, with a modest investment in obtaining ground truth examples, researchers can quickly surpass the performance of an unsupervised algorithm and, instead, approximate the performance of a sophisticated supervised learning algorithm that has access to a large database of ground truth examples using a simple and readily available off-the-shelf linking tool.

## Data and methods

In the present study, we have ground-truth information on true matches, which allows us to report true error rates in order to assess linking performance. Since there may be certain true matches that will be very difficult for any algorithm to find (e.g. if the identifying information is completely different across records), we also estimate a proxy for the “achievable” error rate via an algorithm that has access to all ground-truth examples. To do so, we employ the Name Match Python package and compare the error rate from Name Match to the rates from dedupe. We establish a performance baseline using a popular unsupervised record linkage tool called fastLink.

### Data

Our study protocol was reviewed by the University of Oregon Institutional Review Board (Protocol Number 09052018.005) and was determined to qualify for exemption as per the Common Rule regulations found at Title 45 CFR 46.101(b)(4). This review determined that no informed consent process was applicable for the current study. Our test linking data is a set of identified administrative records on three million charges filed in Oregon courts between 1990–2012, maintained in the Oregon Judicial Information Network (OJIN). The individual records in the OJIN data contain a number of relevant data points: name, date of birth, race, and gender of the defendant; the date the case was filed, a description of the charge, and a unique identification number that links the same defendants across rows in the data set. Around 77% of the sample is White, while Hispanic and Black individuals each makes up around 10% of the sample. Nearly 80% of the individuals are male, and the average age is around 32.6 years (SD = 11.1 years). We link the data using first name, middle initial, last name, date of birth, race, gender and age. We use the unique identifier as our indicator of ground truth to assess whether the algorithms are able to correctly identify links at the person level. Among the records that we can identify as the same person, because they share a unique identifier, 63.7 percent match exactly on name, 90.7 percent match exactly on date of birth, and 59.7 percent match exactly on both name and date of birth (see S1 Appendix for details about how far off non-exact matches are in the data). We collapse the file to the case level, because there is no variation in a person’s identifying information across charges within a case. This leaves us with 1.3 million cases.

### The linking task

The record linkage task we are simulating using these data is a link between a small data set from a research experiment, which we’ll call *E* (one record per person), and a larger administrative data set, *D* (potentially multiple records per person). Our set up mirrors a common linking task in a number of settings, such as linking the names of youth employment program participants to the list of arrestees to examine the crime-reduction effect of employment, or linking the list of election candidates to the list of corporate directors to study if politically connected business had easier access to loans [31, 32]. We further subdivide *E* and *D* into training *E*_*t*_ and *D*_*t*_ and evaluation *E*_*e*_ and *D*_*e*_ portions, since we do not want to evaluate results of a learning algorithm on in-sample data. We also ensure that there is no overlap between the training and evaluation data sets. In the specifications to follow, the size of the training and evaluation sets are the same. Experimental data sets *E*_*t*_ and *E*_*e*_ are always set to 1,000 unique individuals. The administrative data sets vary between 5,000, 50,000, and 200,000 records, where *D*_*t*_ and *D*_*e*_ are the same number of records in each specification. In other words, in every specification, we can think of our objective as attempting to link a person-level experimental data set of 1,000 observations to a case-level data set of either 5,000, 50,000 or 200,000 records.

Finally, we hold the distribution of records per person constant at values reasonably similar to the full data set’s natural distribution.

The distribution of records per person in the administrative data *D* is as Table 1 shows:

**Table 1:** Distribution of records per individual in training and evaluation data.

Records per person	Proportion
1	.50
2	.40
3	.06
4	.03
5	.01

We fix the true match rate, meaning the rate of individuals in the experimental data *E* who have a true match in the administrative data at 50% to facilitate performance evaluation. In other words, 50% of the people in the experimental data set have one or more records in the administrative data set. If our linking algorithm performs perfectly, we would expect to find a link in *D* for the 500 people from *E* that have one. Among these true matches, the share of matches where the records in the experimental data *E* and the administrative data *D* match exactly on first name, last name and date of birth is approximately 50% for both the training and evaluation sets. Meaning that of the 1,000 individuals in *E*, 500 have a record in *D* and 250 of those have a record in *D* that matches the information in *E* exactly on first name, last name, and date of birth.

This particular type of linking task is becoming increasingly common in the social sciences, particularly as it relates to the proliferation of the “low-cost” randomized controlled trial, in which a data set of participants from an intervention is linked to existing administrative databases to capture outcome data in one or more domains [33].

### Running dedupe

The dedupe tool can perform linking tasks using either a traditional record linkage framework, which only considers links between the administrative database *D* and the experimental data *E* or a de-duplication linkage framework in which the records in the experimental data *E* are appended to the administrative data set *D* making one large database with the number of records equal to the sum of the records from *E* and *D*. From there, links are identified via de-duplication meaning that, unlike the record linkage framework, the process considers links within the administrative database *D* and also within the experimental data *E*. To streamline the presentation of results here, we present results for the de-duplication linkage framework, because it tends to have better performance in this context than the traditional record linkage framework. Although the patterns we discuss hold for both kinds of linkage frameworks (see S1 for the traditional record linkage framework results).

Evaluating all possible pairs of records as potential links is often too computationally intensive to be feasible in real-world linking tasks. As a result, record linkage processes begin with a blocking step designed to reduce the number of comparisons required during linking by eliminating those that have low likelihood of being true matches. During blocking, records are organized into groups that consist of broadly similar records according to particular criteria. For example, blocking on birth year would mean that only records with the same birth year will be considered as potential links. The goal is to choose blocking criteria to discard as many nonlinks as possible without discarding true links. Once the data is organized into blocks, the linking task proceeds by assessing pairs of records within blocks to find potential links. This restriction of the comparison space can greatly reduce runtime of the linkage task, but often forces the researcher to make an a priori decision about which blocking rules to impose. Dedupe’s blocking step is part of the active learning process. The optimal blocking rules are part of what is learned via the active learning process, with dedupe learning blocking rules to maximize the share of true pairs that make it past the blocking step. Accordingly there are no a priori decisions that the user has to make about what defines a block before the linking process begins.

This active learning component, which typically requires users to label some number of pairs as either a match, non-match, or unknown, is a critical component of dedupe’s linking process. As previously noted, active learning algorithms work by identifying unlabeled *potential* links that are likely to provide the most information for the training procedure. Dedupe’s active learning step starts by creating four different types of queues, in decreasing order of expected gain in predictive information.

1. Type A: Record pairs that are not blocked together, but have high link probabilities (greater than 0.5)
2. Type B: Record pairs that are blocked together, but have low link probabilities (less than 0.5)
3. Type C: Record pairs that are blocked together and have high link probabilities
4. Type D: Record pairs that are not blocked together and have low link probabilities

**Fig 1:**
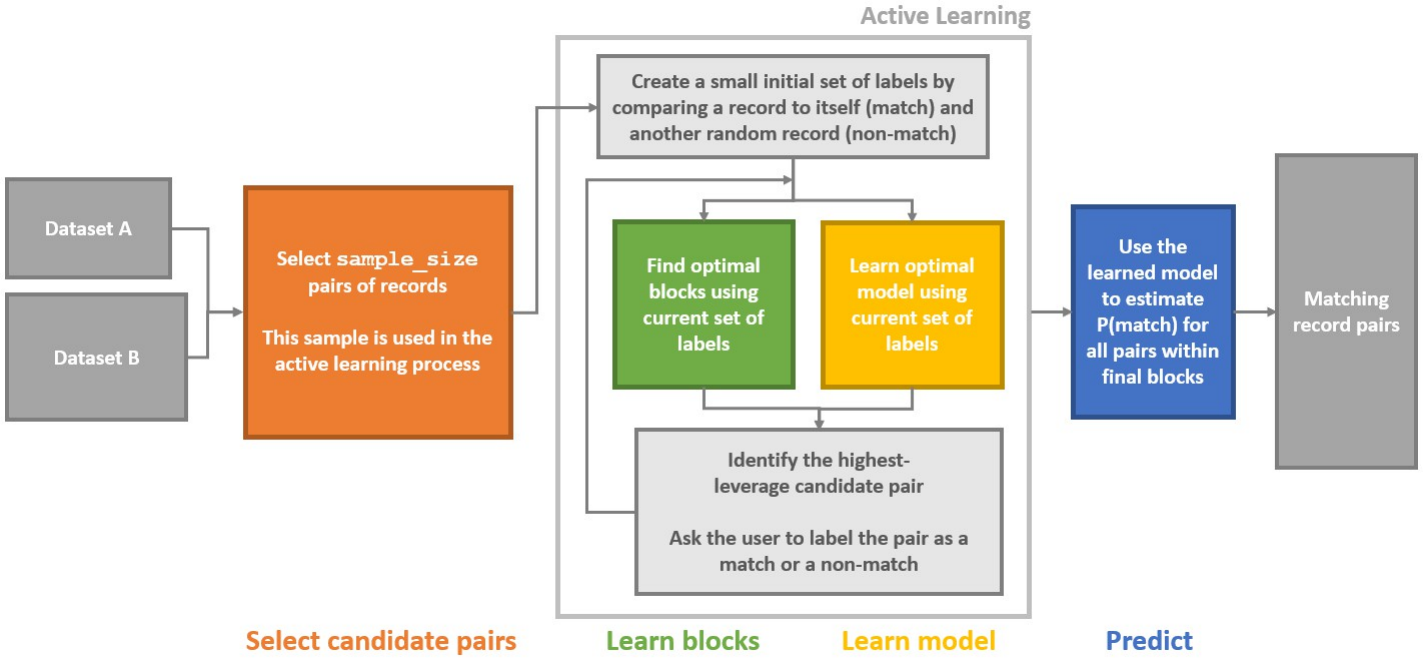
Dedupe’s active learning process.

At each step, dedupe presents the human labeler with a sample from the non-empty queue with the highest priority. For example, if there are no record pairs that meet the criteria for the first queue, then a record pair from the second queue is sampled for human labeling. Predicted probabilities are generated from a regularized linear regression model using the instances labeled up to that point as the outcome. The flowchart below documents dedupe’s process (Fig 1).

We simulate human input by feeding dedupe the labels it requests from ground truth data in order to ensure consistency. By automating the active learning process using ground truth data, we are evaluating dedupe’s performance under optimal conditions, in which there are no errors in the labeling process. In other words, we are assessing dedupe’s performance under the condition that what it learns from the labeling process is entirely correct. The active learning component of dedupe asks the user to label training pairs until it reaches a minimum of 10 positive and 10 negative labels. While dedupe does not limit the training process, the website notes that it should be rare to need more than 50 positive and 50 negative cases to train the algorithm on even the most difficult and messy data sets [34].

There are parameters in dedupe that control the set of pairs that are available during the active learning step of the linking process. The “sample size” parameter determines the number of label-eligible record pairs and the “blocked proportion” parameter influences what share of label-eligible record pairs are similar pairs vs. random pairs. We will show that in the context of our linking task, performance was highly sensitive to the choice of the sample-size parameter. In contrast, our results were not sensitive to variations in blocked proportion. As a result, we fixed blocked proportion at the deduplication framework’s default value of 0.90. A value of 0.90 means that 90% of all the record pairs that the user could be asked to label are records that were similar enough to appear in the same block at least once. The remaining 10% are random pairs of records that, most likely, look nothing alike. Finally, we also make a minor adjustment to the train function. The “recall” parameter is set to 1 by default, meaning that the algorithm must find blocking rules such that 100% of labeled matches are blocked together. We adjusted the recall parameter to.99, because occasionally there are real world cases where an individual actually has two very different looking records. We run dedupe four times for each specification of our linking task in order to observe any variation in linking performance that may occur due to random sampling performed by the algorithm. The version of dedupe used in this paper is v2.0.6, with minor modifications to allow for automated labeling.

### Implementation platform

We ran dedupe with access to 8 Intel Xeon Platinum 8180 CPUs and 700 gigabytes of RAM. One of the benefits of linking using dedupe is that it can be run on a personal laptop and does not require specialized hardware. While we did not conduct an extensive run-time analysis, the relatively short duration of even the longest dedupe run (an average of 134 minutes for a specification with a budget of 1,000, an administrative dataset size of 200,000, and a sample size setting of 150,000), suggests that dedupe’s runtime is a very reasonable investment for the purposes of constructing an analytic data set.

### Benchmarking using a supervised learning algorithm

Since administrative data linking is a noisy task under the best conditions, solely comparing dedupe’s performance to the true link rate is a stringent standard. Accordingly, we also conducted a benchmarking exercise so that we had a point of comparison for dedupe’s performance that was more indicative of what might be possible for a linking algorithm that had access to all the ground truth information, which is likely the best possible performance under real world conditions. To establish this best-case-scenario benchmark, we used a supervised record linkage algorithm, Name Match, to link each combination of experimental dataset *E* and administrative dataset *D*. Name Match was given access to the same data fields dedupe used, plus all of the ground truth information available. We used the Name Match tool’s default settings, including the default blocking scheme which considers two records a potential link if their birthdays are within 2 character edits of each other and their names are reasonably similar. Results are based on Name Match version 1.1.0.

### Benchmarking using an unsupervised learning algorithm

To demonstrate the performance gains achievable through modest investment in generating ground truth labels, we also establish a performance baseline using one of the most popular unsupervised record linkage tools, fastLink. This allows us to understand expected linking performance in a circumstance where none of the ground truth information is available. Specifically, we used fastLink to link each combination of experimental dataset *E* and administrative dataset *D*. We gave fastLink access to the same seven data fields dedupe and Name Match used, but none of the ground truth information. When using fastLink, we chose a blocking scheme such that two records were considered a potential link if their birth years were within one year (window.block) and their last names were loosely similar (kmeans.block with 2 clusters). This is a reasonable blocking scheme and closely resembles the blocking scheme used in Name Match. Otherwise, all of fastLink’s parameters were left to default values. Results are based on fastLink version 0.6.0.

### Evaluation metrics

There are a number of candidate performance metrics we can use to assess the performance of record linking tools. We focus on three evaluation metrics: precision, recall, and total error. Precision and recall are commonly used to evaluate the performance of information retrieval and prediction tasks, because they each focus on a different type of error. In the context of data linkage, high precision indicates fewer false positives. In other words, the algorithm is not linking records that belong to two different individuals. Whereas, recall focuses on false negatives. High recall means that the algorithm is not missing the true links that do, in fact, belong to the same individual. While, ideally, an algorithm has both—high precision and high recall—there is an inherent trade off between the two: fewer false positives typically results in more false negatives, and vice versa. In some cases, the relative costs of false positive and false negative links varies, so it is useful to assess these two measures separately. The total error metric—which is a combination of the false positive rate (FPR) and the false negative rate (FNR)—is also key because the bias introduced by linkage errors is a function of the total linking error [11]. Naturally, to minimize the bias introduced by the linking process, we want to minimize the total error metric.

## Results

### Off the shelf performance

**Fig 2:**
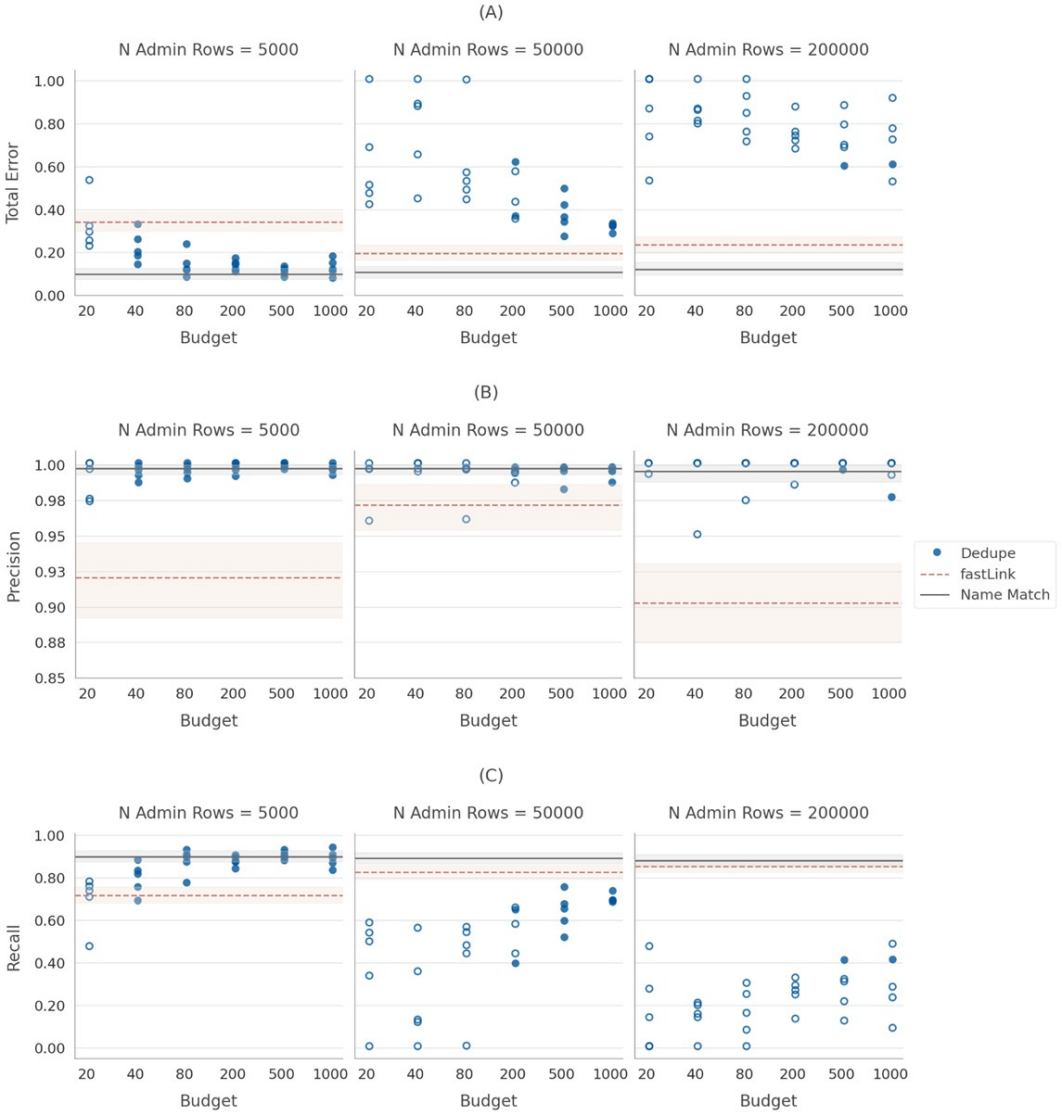
Performance of dedupe, with “off the shelf” setup. A: Total error. B: Precision. C: Recall.

We begin by assessing dedupe’s “off the shelf” performance in the task of linking the experimental data set, *E*, to administrative data sets, *D*, of varying sizes. Fig 2 presents the results of this exercise using dedupe’s deduplication framework to link *E* to *D*. We perform the task with dedupe’s default settings: the “sample size” parameter set to 1,500 label-eligible pairs and blocked proportion set to 0.90. We compare dedupe’s performance to that of an unsupervised learning algorithm (fastLink) and a supervised learning algorithm (Name Match).

In each of the plots, the dashed peach line represents the performance of the unsupervised learning algorithm (fastLink) and the solid black line represents the performance of the supervised learning algorithm (Name Match). In order to generate confidence intervals around algorithm performance, we developed a bootstrapped inference procedure (described in detail in S1 Appendix), the shaded areas on the plots represent the bootstrapped 95% confidence interval. Each of the points represents a separate run of dedupe. (Note that some of the runs had nearly identical performance, such that they are not individually distinguishable in the figure.) The plots in Columns 2 (n = 50,000) & 3 (n = 200,000) of Fig 2 had extraordinarily high total error rates, much higher than the “baseline” performance of the unsupervised learning algorithm and in some cases approaching 100 percent. For example, the best performance for 50,000 admin rows was 33 percent total linking error even after feeding the algorithm 1,000 accurate ground truth labels. The results in Column 3 reveal even larger error rates. Here the best total error rate was 54 percent—for the task of linking an experimental data *E* of 1,000 individuals to an administrative data set *D* containing 200,000 rows with 1,000 accurate ground truth labels. This is a concerning result given that many administrative data sets that might be targeted for linking tasks are quite large. In order to investigate the reason for such high error rates in Fig 2, we next examine the sensitivity of the results to varying each of dedupe’s hyperparameters (i.e., the set of adjustable parameters).

### “Sample size” implications

**Fig 3:**
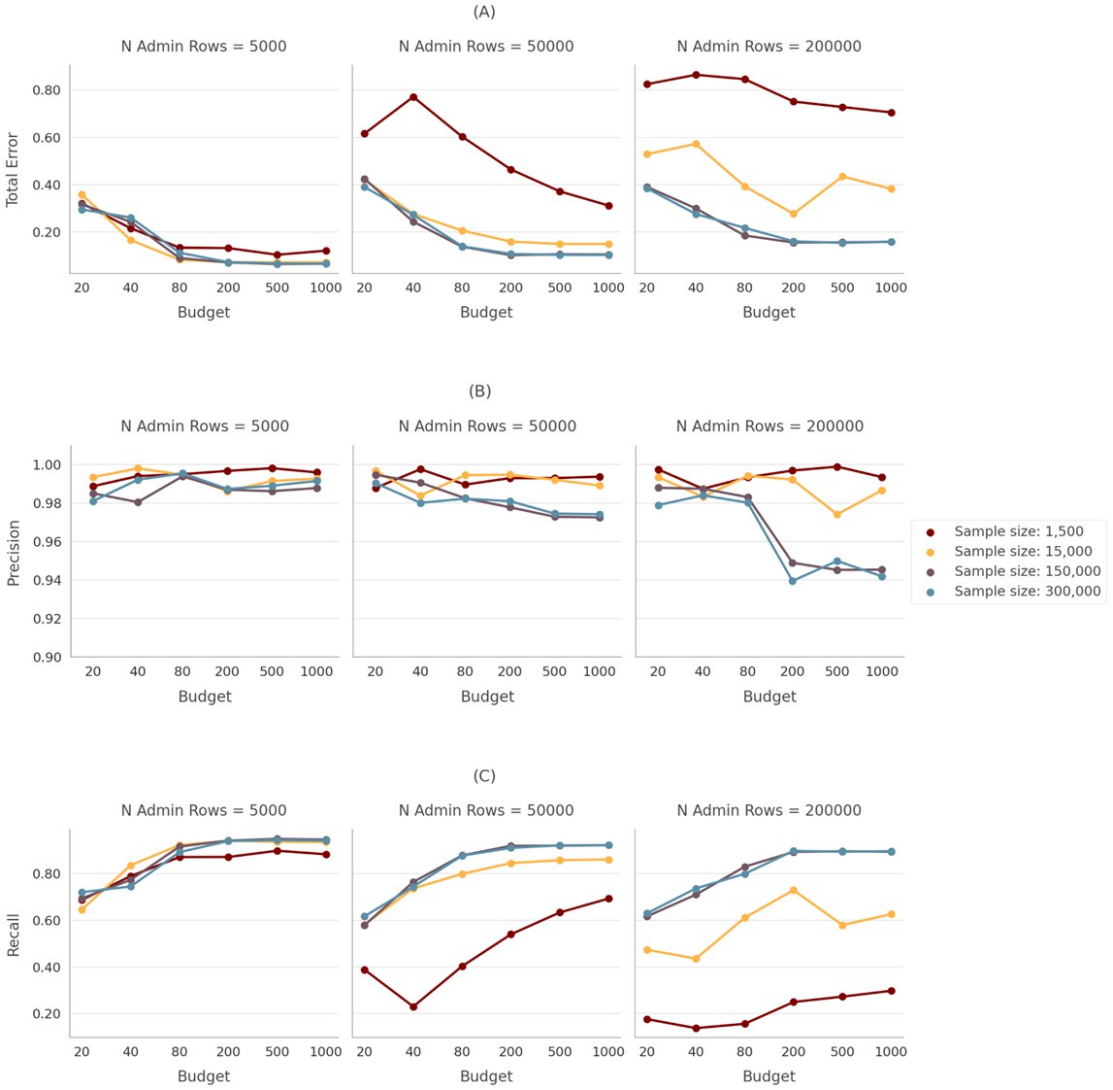
Performance for different sample sizes. A: Total error. B: Precision. C: Recall.

We found that linking performance using dedupe is highly sensitive to the “sample size” parameter as the size of the administrative data set *D* increases. In other words, for larger administrative data set sizes, dedupe needs a larger pool of candidate pairs from which it can select pairs to be labeled during the active learning component of the linking process. Fig 3 shows the performance of the linking task for “sample sizes” of 1,500 (default), 15,000, 150,000 and 300,000 candidate pairs. With respect to performance, all of the sample sizes performed similarly with respect to precision (Fig 3, Panel B). While precision performance is not very sensitive to sample size, increasing the number of candidate pairs to which dedupe has access when it is looking for candidates to label has large effects on recall performance (Fig 3, Panel C). As Panel C, Column 3 shows, recall never exceeded 40 percent on average even when we provided accurate labels for 1,000 of the 1,500 candidate pairs. By contrast, for all three specifications, a sample size of 300,000 candidate pairs performed well on both precision and recall and, by extension, total error. The “sample size” induced variation in dedupe’s performance is driven by the availability of “high value” candidate pairs that, when labeled, provide the most predictive information for the algorithm. These “high value” candidate record pairs are relatively rare, and therefore increasing the sample size parameter allows dedupe’s active learning step access to more “high value” pairs to present for labeling (see S1 Appendix for a detailed analysis of the types of candidate pairs selected for labeling at various sample sizes).

We performed a bootstrap inference procedure to test whether adjusting the sample size parameter has a significantly better performance than default dedupe (see S1 Appendix for details). For each bootstrap sample and performance metric, we take the difference between the performance metric values for “sample size”-adjusted dedupe and default dedupe. We perform this process for each linking specification considered, i.e. size of dataset D and the label budget provided to dedupe. Thus, for each linking specification, we have a bootstrap distribution of the difference in performance metrics between adjusted and default dedupe. We consider the performance of “sample size”-adjusted dedupe to be significantly better than default dedupe if the difference in performance is less than zero at the 95th percentile of the empirical distribution for total error.

We find that changing dedupe’s sample size parameter to 150,000 potential pairs results in significantly better performance than the default version of dedupe in the majority of comparisons. In Table 2) the median column compares the total error rates for the median run of “sample size”-adjusted dedupe and default dedupe. The worst-case column compares the best run of default dedupe to the worst run of sample size adjusted dedupe. The asterisks in the table indicate that the difference in performance between “sample size”-adjusted dedupe and default dedupe is less than zero at the 95th percentile of the empirical distribution of differences in total error rate. The table also presents the percentage of comparisons for which “sample size”-adjusted dedupe was a significant improvement over default dedupe. The benefit to increasing the sample size parameter is most notable when the administrative database *D* is relatively large. When *D* has 50,000 rows, we see significant improvements when we adjust the sample size parameter, where the number of labels is 80 or more. In that case adjusting the sample size results in a significantly lower total error rate in 60–100% of possible comparisons depending on the number of labels that are used. The benefits to adjusting dedupe’s sample size parameter are even more dramatic when the size of the administrative database *D* is 200,000 rows. In those tests, the “sample size”-adjusted version of dedupe performed significantly better than default dedupe (with respect to total error) for between 80–100% of possible comparisons. However, there are costs to the speed of the process by substantially increasing sample size. To balance run time costs and performance, we shifted to a sample size of 150,000 for all specifications.

**Table 2:** Statistical comparisons of total error rate for “sample size”-adjusted dedupe and default dedupe.

		Comparison		
*D*	Budget	Median	Worst-case	Share significant
5000	20			24%
	40			24%
	80	*		64%
	200	*	*	100%
	500	*		80%
	1000	*		80%
50,000	20	*		80%
	40	*	*	100%
	80	*	*	100%
	200	*	*	100%
	500	*	*	100%
	1000	*	*	100%
200,000	20	*		96%
	40	*	*	100%
	80	*	*	100%
	200	*	*	100%
	500	*	*	100%
	1000	*	*	100%

The median column compares the performance of the median runs of each algorithm. The worst-case column compares the best run of default dedupe to the worst run of “sample size”-adjusted dedupe.

* Indicate significant difference in total error rate between “sample size”-adjusted dedupe and default dedupe. Share significant indicates the percentage of comparisons where “sample size”-adjusted dedupe was a significant improvement over default dedupe.

### Main results

**Fig 4:**
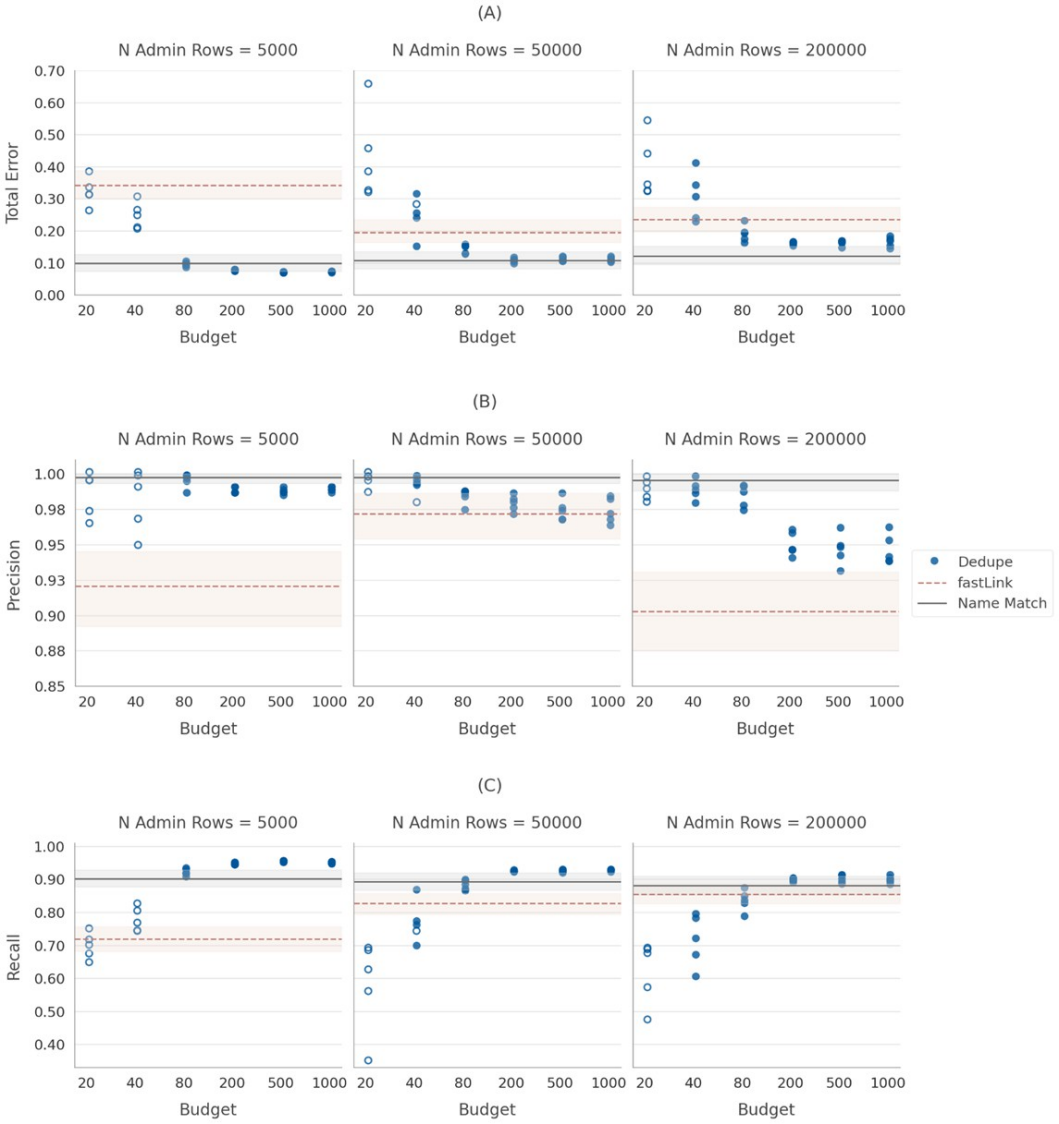
Performance of dedupe under the deduplication framework, sample size 150,000. A: Total error. B: Precision. C: Recall.

Fig 4 presents the performance of our linking task with the larger “sample size.” A couple important findings emerge. First, when we look at the plots in Fig 4 we see that many more of the dots are filled in compared to the dots on the plots in Fig 2, indicating that the runs were much more likely to meet the baseline training threshold of 10 positive and 10 negative links during the labeling process when dedupe is given access to a larger pool of candidate links during the labeling process. However, we also see that even conditional on meeting the training criterion of 10 positive and 10 negative labels dedupe’s performance improves when we provide more ground truth labels *up to a point* when it starts to flatten out around 200 labels. We see from the figure that there are diminishing returns to providing ground truth labels after approximately 200 labels. We found that the performance improvement levels off after 200 labels, because after that point there are fewer and fewer “high value” candidate pairs (we provide a detailed demonstration of this relationship in the S1 Appendix). This is a key result because it means that in linking contexts when there are no pre-existing ground truth labels available, linking using active learning performs well with reasonable investments in ground truth labels. The performance improvements are largely driven by improvements in recall performance.

The result is even more remarkable put in context. Fig 4 shows the performance of the unsupervised learning algorithm, fastLink, as a baseline and the performance of the supervised learning algorithm, Name Match, as a benchmark for the linking performance we might expect under the best case scenario of access to a large amount of ground truth information. For all of the administrative data set sizes we tested, we are able to outperform the popular unsupervised learning algorithm and achieve performance similar to (or better than) a supervised learning algorithm by using dedupe’s active learning algorithm and an investment in providing approximately 200 ground truth labels (or fewer in some circumstances). To formally differentiate between the performance of the algorithms, we used the same bootstrap inference procedure (described in detail in S1 Appendix). Table 3 presents the results of these comparisons. In Table 3 the median column compares the total error rates for the median run of “sample size”-adjusted dedupe to fastLink (Panel A) or Name Match (Panel B). The worst-case column compares the best run of fastLink to the worst run of “sample size”-adjusted dedupe (Panel A) or the best run of Name Match to the worst run of “sample size”-adjusted dedupe. The asterisks in the table indicate that the difference in performance between “sample size”-adjusted dedupe and fastLink or Name Match is less than zero at the 95th percentile of the empirical distribution of differences in total error rate. The table also presents the percentage of comparisons for which “sample size”-adjusted dedupe was a significant improvement over fastLink (Panel A) and for which Name Match was a signficant improvement over “sample size”-adjusted dedupe (Panel B). In these tests, dedupe outperforms fastLink with respect to the total error rate for nearly every type of comparison tested, including all but one instance comparing dedupe’s worst performance to fastLink’s best performance. Indeed, for this particular linking task, in all but two scenarios (label budget = 20, *D* = 5, 000 & *D* = 50, 000), dedupe significantly outperformed fastLink in 100% of possible comparisons.

**Table 3:** Statistical comparisons of total error rate between “sample size”-adjusted dedupe with fastLink and Name Match.

**Panel A: “Sample size”-adjusted dedupe vs. fastLink**				
		Comparison		
*D*	Budget	Median	Worst-case	Share significant
5000	20			24%
	40	*		84%
	80	*	*	100%
	200	*	*	100%
	500	*	*	100%
	1000	*	*	100%
50,000	20			0%
	40			20%
	80	*	*	100%
	200	*	*	100%
	500	*	*	100%
	1000	*	*	100%
200,000	20			0%
	40			0%
	80	*		80%
	200	*	*	100%
	500	*	*	100%
	1000	*	*	100%
**Panel B: Name Match vs. “sample size”-adjusted dedupe**				
		Comparison		
*D*	Budget	Median	Worst-case	Share significant
5,000	20	*	*	100%
	40	*	*	100%
	80			0%
	200			0%
	500			0%
	1000			0%
50,000	20	*	*	100%
	40	*	*	100%
	80	*	*	64%
	200			0%
	500			0%
	1000			0%
200,000	20	*	*	100%
	40	*	*	100%
	80	*	*	100%
	200	*	*	96%
	500	*	*	80%
	1000	*	*	76%

* Indicate significant difference in total error between the algorithms.

We also tested whether the total error rates for dedupe and Name Match were statistically distinguishable from one another. However, unlike with the previous comparisons, where an analyst will want to be able to statistically distinguish between the performance of each of the comparisons, here, the most salient question is whether dedupe can return total error rates that are not statistically significantly different from those returned by Name Match. For administrative database sizes 5,000 and 50,000, provided dedupe has at least 80 labels, dedupe’s performance is indistinguishable from Name Match’s performance. When the administrative database is 50,000 rows, Name Match performed significantly better than dedupe only when we compared the worst performing run of dedupe to the best performing run of Name Match and, even then, in less than one-third of the possible comparisons when we provided dedupe with at least 200 labels. For the largest administrative database size we tested (200,000 rows) we see that although the results are not quite as dramatic, dedupe still performs to a level that is statistically indistinguishable from Name Match in more than 40% of possible comparisons when we provide at least 200 labels. This is a very encouraging result. With a few small adjustments to an open source package, researchers who have data linking needs but little specific linkage expertise can leverage active learning and a relatively small investment in providing ground truth labels to achieve remarkable performance even if their data is quite messy.

Importantly, we are not suggesting that active learning with a modest number of labels approximates the linking performance we might expect from a supervised linking process in all linking cases. However, in this specific case, we are able to approximate the performance of a supervised learning algorithm with a small number of ground truth labels. While the case we set up is specific, it is also a very common linking task in empirical social science.

## Discussion

In this paper, we investigate the value of ground-truth examples for linking performance. Using a popular open-source record linkage algorithm, *dedupe* [30], we explore how the payoff to investing in ground truth labels varies based on the context and the difficulty of the linking task, such as the size of the training dataset and the size of the datasets involved in the linking task itself. We show that in many applications, only a relatively small number of tactically-selected ground-truth examples are sufficient to obtain most of the achievable gains. With a modest investment in ground truth, researchers can approximate the performance of a sophisticated supervised learning algorithm that has access to a large database of ground truth examples using simple and readily available off-the-shelf tools. Furthermore, our results show that making a modest investment in labeling yields significant gains over methods that do not leverage ground truth information at all.

Of course, it is important to discuss some of the limitations of our work. First, the number of labels required to maximize achievable performance is likely to grow with the size of the administrative dataset being linked. Our results speak to linking scenarios using databases that have fewer than 200,000 observations. Next, as we show in this paper, linkage performance is related to the difficulty of the matching task. We have explored one particularly common and important type of linking task in this paper, matching individuals when we have access to full name and date of birth information. To the extent other types of linkages are more or less difficult, that would have implications for the number of ground-truth labels needed to obtain maximum achievable performance. To that end, further avenues for research would include exploring other types of linkages: not having access to name and/or dob in full, or non-person linking such as linking location or organization names. Third, we explored how many labels are required to get back to overall levels of performance. However, there may still be differences in performance by subgroups such as by race or gender.

## Conclusion

Most often, social scientists need to link data without access to ground truth or to a collaborator who can implement “state of the field” machine learning linking techniques. Although many applied research projects could benefit from implementing processes like active learning to perform record linkage tasks, there is relatively little practical guidance available. Often applied social science researchers consider record linkage to be a pre-processing step, rather than an early-stage component of their analytic plan. However, as prior work has demonstrated, record linkage errors can increase bias and diminish statistical power [11]. To that end, our paper serves as a guide for how researchers can benefit from the power of record linkage without learning data scientific methods from scratch. An added benefit is that using a publicly available linking tool like dedupe promotes transparency and replicability since the underlying code is freely available, which is particularly important for applied researchers who have little formal training in data scientific methods. Our ultimate goal is to reinforce the point that data linking is an important component of empirical analysis with implications for how social scientists understand their analytic results and to demonstrate that in many cases relatively minor investments in ground truth labeling using an open-source package can reduce the consequences of linking error and improve the validity of empirical social science research.

## Supporting information

S1 Appendix Technical appendix. (PDF)
