# Supplementary material for: No ground truth? No problem: Improving administrative data linking using active learning and a little bit of guile
Source: PLoS One. 2023 Apr 4;18(4):e0283811. doi: 10.1371/journal.pone.0283811 (PMC10072450; doi:10.1371/journal.pone.0283811)

S1 Appendix. Technical Appendix

A Supplemental Data Description

Table A.1: Supplemental Data Description The table below presents the share of the data that exact matches on name, date of birth, or both. For those records that do not match on name, the table presents the share of records that are off by increasing numbers of letters, and for those records not matching exactly on date of birth the table presents how many numbers the records are off by. The numbers could be any of the digits in the DOB.

Overall match	Specific match	Percentage
Matching SID	Exact match on name	63.7%
	Exact match on DOB	90.7%
	Exact match on name and DOB	59.7%
For ones not exactly matching on name	Off by 1 letter	16.6%
	Off by 2 letters	10.9%
	Off by 3 letters	11.9%
	Off by 4 letters	12.0%
	Off by 5 letters	11.8%
	Off by 6 letters	10.8%
	Off by 7 letters	6.7%
	Off by 8 letters	4.7%
	Off by 9 letters	2.4%
	Off by 10+ letters	12.1%
For ones not exactly matching on DOB	Off by 1 numbers	54.0%
	Off by 2 numbers	15.8%
	Off by 3 numbers	8.2%
	Off by 4 numbers	10.6%
	Off by 5+ numbers	11.0%

B Types of Candidate Pairs By Sample Size

As we describe in the paper, dedupe's active learning process starts by creating four different types of queues, in decreasing order of expected gain in predictive information.

1. Type A: Record pairs that are not blocked together, but have high link probabilities (greater than 0.5)

2. Type B: Record pairs that are blocked together, but have low link probabilities (less than 0.5)

3. Type C: Record pairs that are blocked together and have high link probabilities

4. Type D: Record pairs that are not blocked together and have low link probabilities

By way of example, the figure below shows the share of pairs labeled by type for a budget of 200 labels. At the default sample size of 1,500 potential record pairs, there are very few Type A pairs available and the number of Type B pairs available decreases sharply as the size of the administrative data set increases. dedupe resorts to asking for labels for Type C and D candidate pairs when we use the default sample size for administrative data set sizes of 50,000 and 200,000. In contrast, the larger sample sizes provide dedupe with a greater share of Type A candidate pairs available for labeling. We can also see from the figure that there are similar shares of Type A labels at 150,000 as there are at double that size (300,000). Further, increasing the sample size parameter results in negligible labeling of Type C or D candidate pairs.

Figure B.1: Share of Labeled Pairs By Type and Sample Size The figure shows the share of candidate pairs by type for a budget of 200 labels. Each panel shows the share of each type of label as indicated for sample size 1,500, 15,000, 150,000 and 300,000 at each of three dataset sizes: 5,000, 50,000 and 200,000. Each symbol on the plots represents the average share of each pair type over nine runs of dedupe.

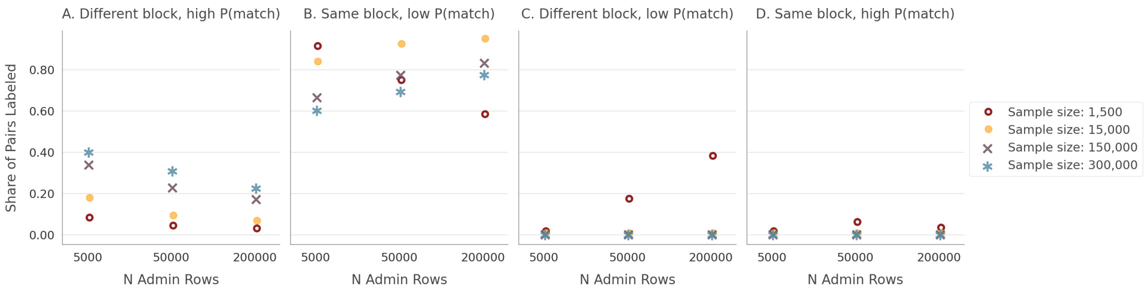

C Bootstrapped Inference Procedure

We developed a bootstrapped inference procedure that allows us to formally test the difference in performance between sample size adjusted dedupe and three comparison algorithms: default dedupe, fastLink, and Name Match. To do so, we generate distributions of the difference in performance between relevant pairs of algorithms using the following bootstrap procedure.

We begin by bootstrapping the experimental dataset E 1,000 times. For each of the 1,000 bootstrap samples E_s, we compute the performance metrics of linking dataset E_s to the administrative dataset D using each algorithm (sample size adjusted dedupe, default dedupe, fastLink, and Name Match). Comparing the performance metrics of sample size adjusted dedupe to the performance metrics of the comparison algorithms for each bootstrap sample gives us the empirical distribution of the difference in performance between linking algorithms. We complete this process for each linking context considered, i.e., size of administrative database D and the label budget provided to dedupe.

These empirical distributions of performance differences allow us to measure statistical significance for three important questions:

- Does sample size adjusted dedupe perform better than default dedupe?
- Does sample size adjusted dedupe perform better than fastLink?
- Does Name Match perform better than sample size adjusted dedupe?

We consider the performance of "sample size"-adjusted dedupe to be significantly better than default dedupe if the difference in performance is less than zero at the 95th percentile of the empirical distribution for total error.

As described in Results section of the main manuscript, we ran each linking algorithm five times for each linking specification in order to understand the variability of the different linking tools on a given dataset. Because of this, we are able to generate 25 measures of significance for each combination of sample size adjusted dedupe and comparison algorithm. In other words, we can determine whether there is a significant difference in performance

between each of the five sample size adjusted dedupe runs and each of the five comparison algorithm runs (yielding 25 total significance measures). In Tables 2 and 3 of the main manuscript, we report the share of significance tests for which there was a significant difference in total error between sample size adjusted dedupe and comparison algorithms default dedupe, fastLink, and Name Match.

D Performance Improvement Analysis

The figure below shows that when we increase the budget beyond approximately 200 labels, dedupe runs out of "high value" Type A pairs where the records come from different blocks but have a high match probability (above 0.5) and the remaining labels end up being lower value Type B pairs, which come from the same block and have low match probability (below 0.5). Panel B in the figure shows that for larger label budgets, most of the labels are coming from Type B pairs, meaning that increasing the label budget above 200 yields very few additional "high value" labels. The return on labeling investment diminishes at that point.

Figure D.1: Count of Pairs Labeled By Budget The figure presents the average number of pairs labeled by type for budgets of 20, 40, 80, 200, 500, and 1,000 labels for varying data set sizes of 5,000, 50,000 and 200,000. Panel A shows the number of Type A pairs labeled where the records come from different blocks with high match probability and Panel B shows the number of Type B pairs labeled which come from the same block with low match probability. Each figure on the plot represents the average from nine runs of dedupe all with sample size parameter 150,000.

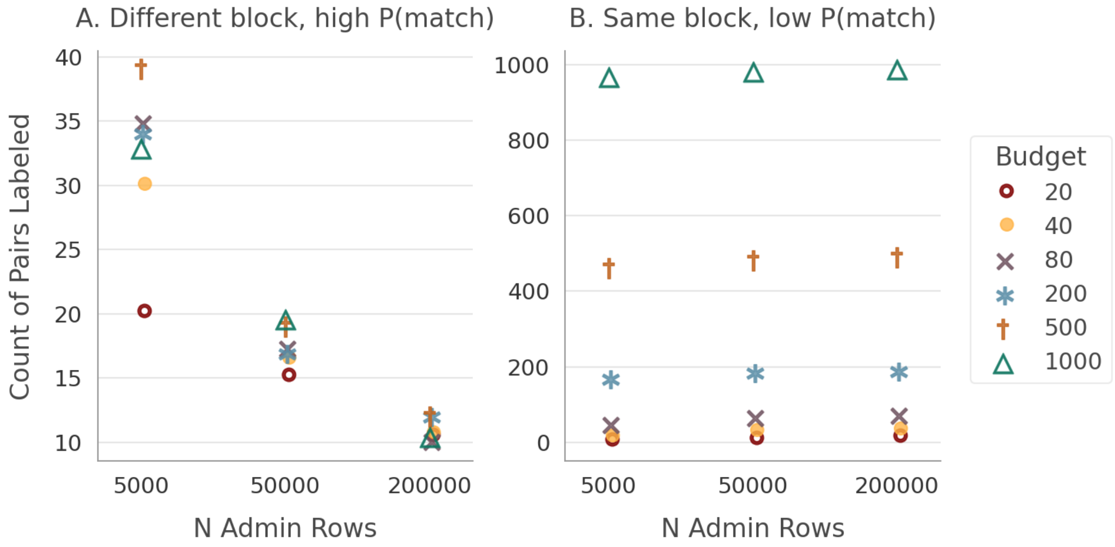

Supplement: S1 Appendix — (PDF) [file pone.0283811.s001.pdf]
